# A Rare and Interesting Presentation of Diabetic Ketoacidosis Caused by Native Aortic Valve Endocarditis Complicated by Aortic Root Abscess Resulting in Multiple Septic Emboli Causing Bowel Ischemia and Splenic Infarction

**DOI:** 10.7759/cureus.29254

**Published:** 2022-09-17

**Authors:** Vijay C Vinod, Umme Farhana, Scott Waring, Gideon Mlawa

**Affiliations:** 1 Medicine, Barking, Havering and Redbridge University Hospitals NHS Trust, London, GBR; 2 Internal Medicine and Diabetes and Endocrinology, Barking, Havering and Redbridge University Hospitals NHS Trust, London, GBR

**Keywords:** latent autoimmune diabetes in adults, superior mesenteric artery thrombosis, bowel ischemia, splenic infarction, septic emboli, aortic root abscess, infective endocarditis, diabetic ketoacidosis

## Abstract

Diabetic ketoacidosis (DKA) is a serious life-threatening complication of diabetes, often precipitated by infection. Infective endocarditis (IE) is a serious precipitating factor for DKA, especially in patients with a previous cardiac surgery or valvular pathology. IE can be further complicated by life-threatening embolic events, which could be fatal if not detected and managed early and effectively. Our patient is a 54-year-old diabetic who presented with DKA precipitated by native aortic valve endocarditis complicated by an aortic root abscess, which was further complicated by septic emboli to the splenic artery and superior mesenteric artery leading to splenic infarction and bowel ischemia, respectively. To our knowledge and as per the literature reviewed, no case has been reported in a single patient who presented with DKA precipitated by IE complicated by aortic root abscess and multiple septic emboli resulting in bowel ischemia and splenic infarction.

## Introduction

Diabetic ketoacidosis (DKA) is a life-threatening condition with significant morbidity and mortality if not managed effectively. Infective endocarditis (IE) is a serious precipitating etiology with high mortality if not detected early [1]. Aortic root abscess may complicate aortic valve endocarditis, which can be exacerbated by a fistula or a pseudoaneurysm [2]. A serious complication of IE is septic embolism into the systemic circulation, which increases the morbidity and mortality further when multiple septic emboli are present [3].

Our patient presented with acute DKA precipitated by infection. Initially, the source was felt to be urosepsis due to cloudy urine accompanied by elevated inflammatory markers. The presence of a murmur along with previous cardiac history diverted our attention to a possible diagnosis of IE, which was supported by a bedside transthoracic echocardiogram (TTE). Transesophageal echocardiogram (TEE) confirmed aortic valve vegetation complicated by aortic root abscess with fistulation. His condition was further complicated by multiple septic emboli to the superior mesenteric artery (SMA) and splenic artery resulting in acute mesenteric bowel ischemia with necrosis and splenic infarction, respectively. Embolic phenomenon in this patient could be from the vegetation or the aortic root abscess. To our knowledge and as per the literature reviewed, no such case has been reported of a patient presenting with DKA precipitated by IE complicated by aortic root abscess and multiple septic emboli.

## Case presentation

A 54-year-old male patient presented to the emergency department with a three-day history of lethargy, reduced oral intake, and nausea. No other symptoms were reported. His medical history included type 2 diabetes, hypertension, chronic obstructive pulmonary disease, and thalassemia trait. He had also undergone aortic valve sparing, aortic root plus ascending aorta replacement surgery due to aortic root disease with severe aortic regurgitation five years ago. There was also a recent history of recurrent DKA. His regular medications were metformin, sitagliptin, bisoprolol, aspirin, and atorvastatin.

On examination, the patient was not in acute discomfort, nor did he complain of any pain. His initial vital signs recorded in the emergency department were as follows: blood pressure of 139/78 mm Hg, heart rate of 97 beats/minute, regular, respiratory rate of 17breaths/minute, temperature of 36.8°C, and SpO_2_ of 98% on room air. Cardiovascular examination revealed an early diastolic murmur on auscultation. Respiratory system and abdomen examination was unremarkable. An arterial blood gas (ABG) was performed to assess for DKA. ABG showed pH of 7.15, HCO_3_ of 11.6 mEq/L, base excess of 16.7 mmol/L, blood glucose of 27 mmol/L, ketones of 6.0 mg/dL, lactate of 5.1 mmol/L. A diagnosis of DKA was established and the patient was started on fixed-rate insulin as per the hospital DKA treatment protocol guidelines. The patient was catheterized showing cloudy urine with sediment.

His initial blood tests showed a white blood cell count of 16.9 x 10^9^/L (reference range: 3.8-11 x 10^9^/L), neutrophil of 15.4 x 10^9^/L (reference range: 2-7.5 x 10^9^/L), C-reactive protein of 281 mg/L (reference range: 0-5 mg/L). No peripheral stigmata of endocarditis were found on examination. Chest X-ray did not reveal any signs of consolidation or evidence of COVID-19 pneumonitis. COVID-19 polymerase chain reaction test was negative. Urosepsis was diagnosed as the precipitating factor for his DKA, and he was started on intravenous amoxicillin and gentamycin as per the adult antimicrobial guide of the hospital trust after blood and urine cultures were sent. However, in view of his cardiac history and a present murmur, a strong possibility of endocarditis was considered, and therefore three sets of blood cultures and a TEE was requested. Reversal of the laboratory and clinical signs of acidosis was observed within the first 24 hours following hospital admission. The patient was positive for anti-gliadin antibodies and negative for islet cell antibodies, which established a diagnosis of latent autoimmune diabetes in adults (LADA). His metformin and sitagliptin were stopped, and he was commenced on a subcutaneous insulin regime.

**Figure 1:**
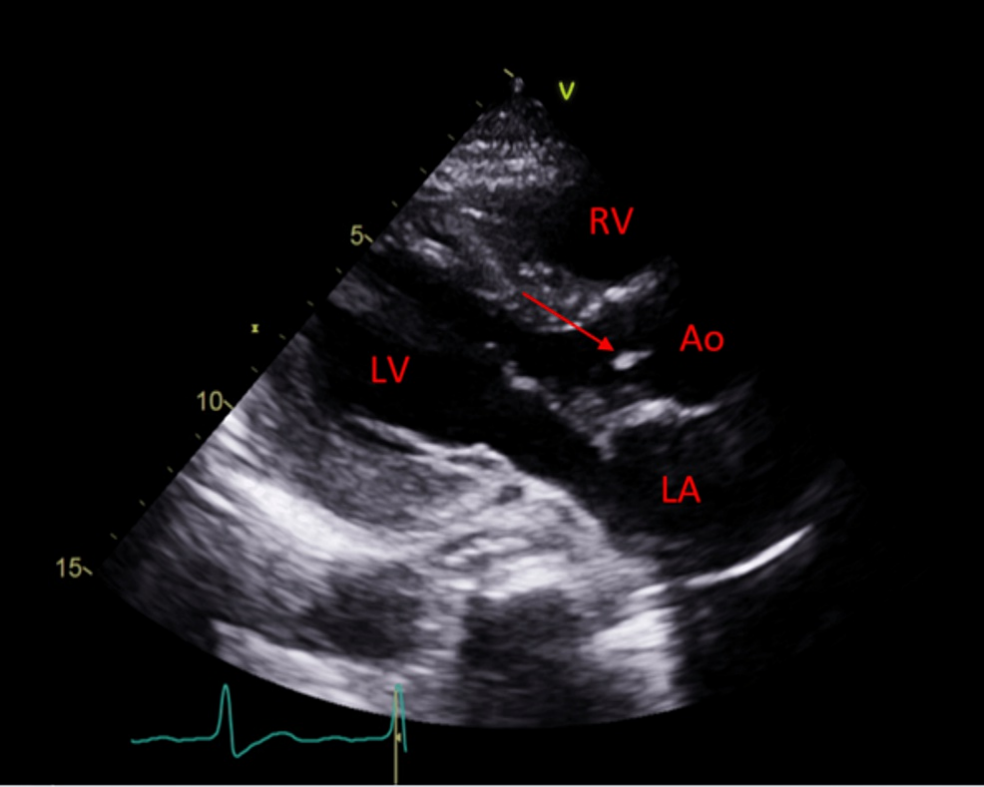
Transthoracic echocardiogram with parasternal long-axis view depicting an echogenic mobile structure tethered to the aortic valve.

**Figure 2:**
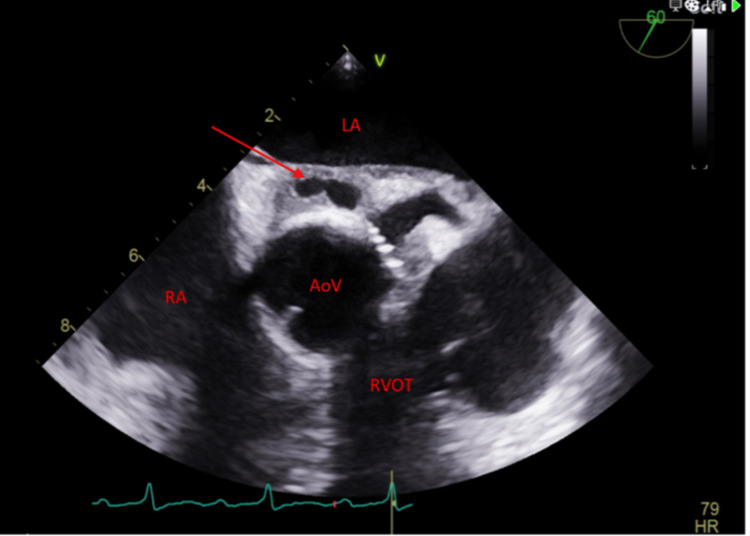
Transesophageal echocardiogram with midesophageal aortic valve view depicting aortic valve with aortic root abscess marked by a red arrow, with an echo-lucent channel centrally. Profound first-degree heart block is also observed. AoV, aortic valve; Ao, aorta; RA, right atrium; LA, left atrium; RV, right ventricle; LV, left ventricle

On the following day, the patient complained of palpitations. Electrocardiogram (ECG) showed normal sinus rhythm with prolonged PR interval of 255 milliseconds (first-degree AV block). Urgent TTE (Figure 1) was conducted on the same day, which showed native aortic valve vegetation at the left ventricular outflow tract (LVOT) region toward the base of the anterior mitral valve leaflet. This was further confirmed by a TEE (Figure 2), which also demonstrated evidence of aortic root abscess and native aortic valve with prolapse left coronary cusp causing severe eccentric aortic regurgitation. Intravenous antibiotics (vancomycin, rifampicin, and gentamycin as per the adult antimicrobial guide of the hospital trust) were initiated for IE.

**Figure 3:**
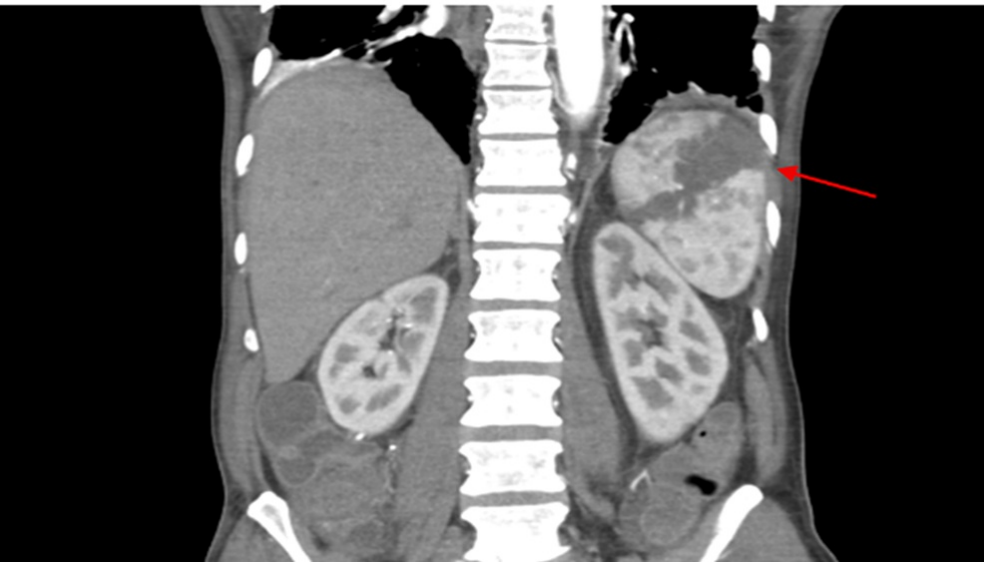
Coronal plane contrast CT of the abdomen and pelvis, with a red arrow highlighting splenic infarction.

**Figure 4:**
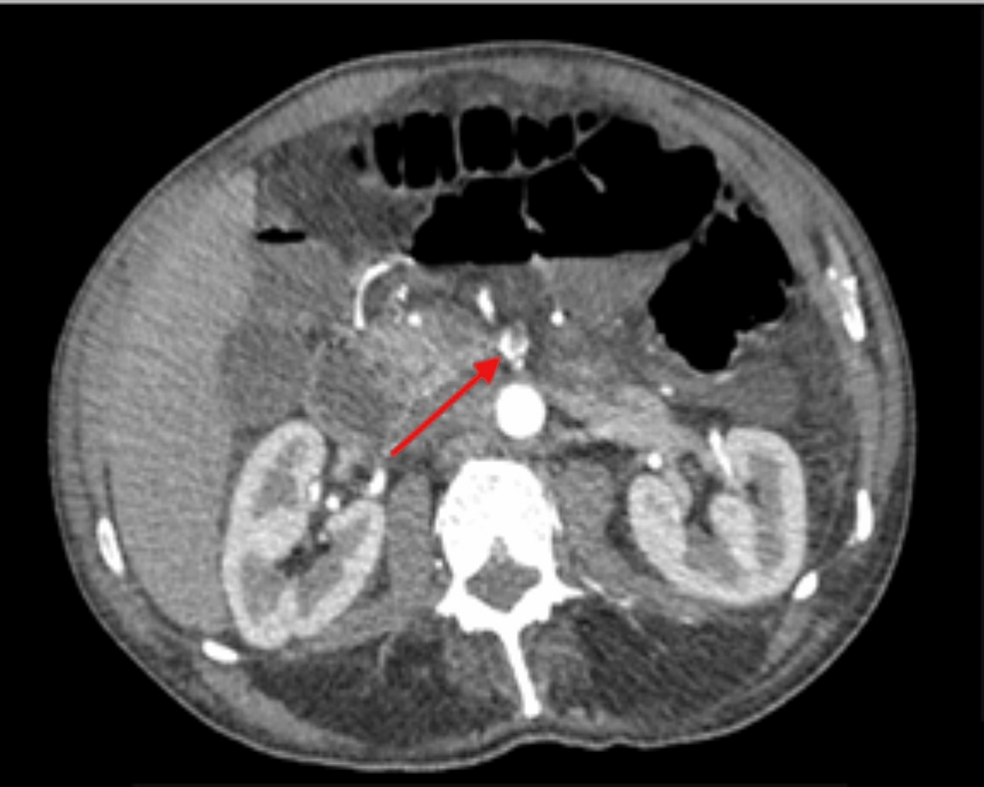
Transverse plane contrast CT of the abdomen and pelvis, with a red arrow depicting partial thrombosis of the superior mesenteric artery. Distended, fluid-filled bowel loops are also visualized.

On the same day, the patient became septic while complaining of abdominal pain. On examination, his abdomen was tender and mildly distended, with absent bowel sounds on auscultation. An urgent computed tomography (CT) scan of the abdomen and pelvis was performed (Figures 3, 4), which showed small bowel obstruction, splenic infarction, superior mesenteric vessel “whirl” sign, and thrombosis of the SMA. No bowel ischemia was reported. A CT angiogram (CTA) was arranged prior to surgical intervention. Treatment dose enoxaparin was started to treat the SMA thrombosis.

**Figure 5:**
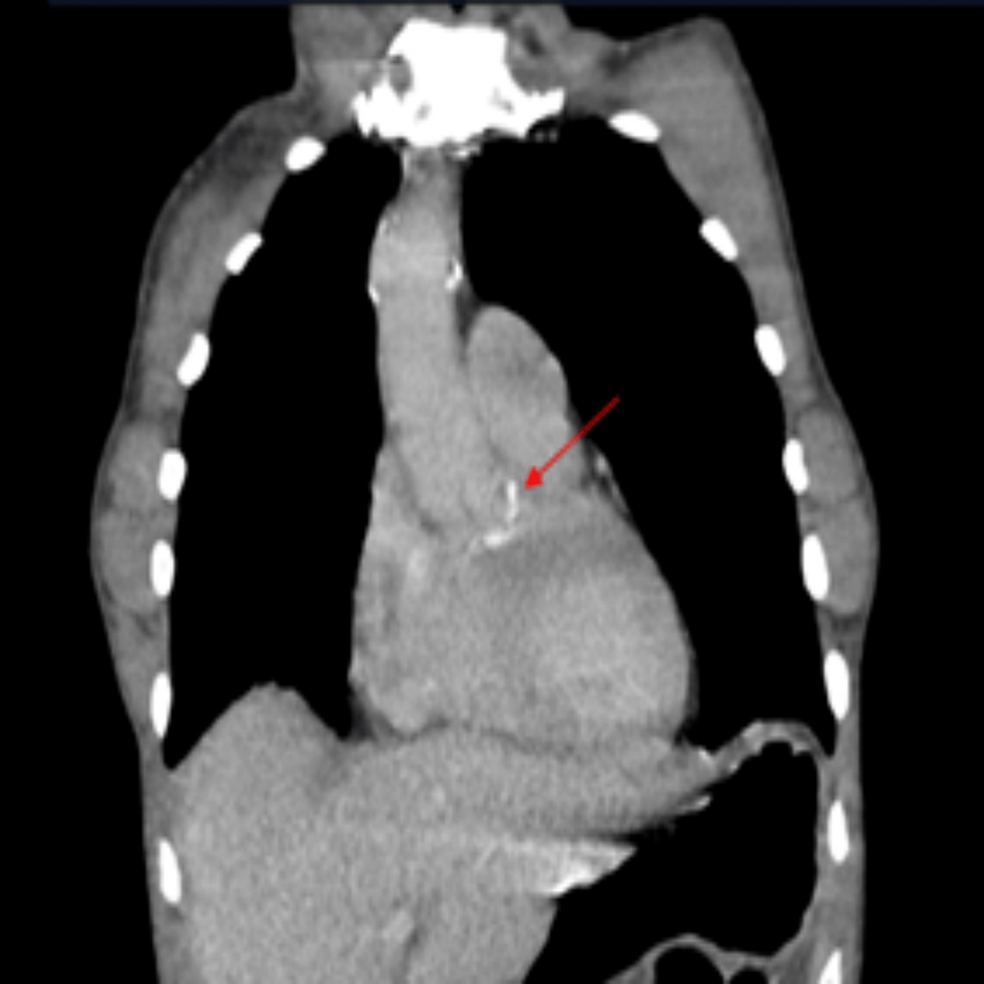
CT aortogram in the coronal plane, with red arrow highlighting site of aortic abscess.

CTA was performed the following day, which demonstrated an acute SMA thrombosis, splenic infarction with a normal enhancement along the splenic artery, and small bowel obstruction secondary to an ileocecal mass. It also showed a 3.4×1.5 cm contrast opacified cavity at the aortic root inseparable from the aortic valve left coronary cusp, suggestive of aortic root abscess (Figure 5). Exploratory laparotomy was undertaken, which revealed an ischemic jejunum and caecum but was salvageable, and therefore bowel resection was not performed. The abdomen was closed temporarily with a drain in situ and planned for a relook surgery in 48 hours. The patient was continued on a heparin infusion.

The results of four sets of blood cultures showed no growth. Local microbiology input suggested to look for signs of dental abscess as cultures were negative; however, no dental abscess or infection was found. Urine culture also showed no growth. Re-look laparotomy surgery was performed after 48 hours, which found a gangrenous 1 cm segment of the small bowel which was 50 cm away from the duodenojejunal junction. Small bowel resection and primary anastomosis were performed. The patient was moved to the intensive care unit for post-operative recovery. A TEE was performed two weeks after admission, which showed evidence of a large echo-lucent channel measuring 1.29×3.81 cm at the level of the sinus consistent with an aortic root abscess or a pseudoaneurysm.

The patient remained at our hospital for over three weeks prior to transfer to a tertiary cardiac center for further surgical management. The patient underwent mechanical aortic valve replacement and drainage of the aortic root abscess with bovine patch repair. We have since followed up with this gentleman and he continues to do well with regular follow-up.

## Discussion

DKA is a medical emergency. DKA has an annual incidence of 3.6% in type 1 diabetics in the UK, and 6% of type 1 diabetics present to the hospital with DKA [4]. Though not common in type 2 diabetic patients, DKA is still diagnosed in daily clinical practice and may also be the first clinical presentation to the healthcare facility [5]. Infection is one of the common triggering or precipitating factors for DKA. It is particularly important to identify the underlying etiology or the source of infection for a better clinical outcome.

IE still poses a great clinical challenge in this modern world, both for early detection and treatment. IE has an extremely high mortality rate of around 30% if left untreated or not diagnosed early [1]. IE may affect the native as well as prosthetic valves of the heart. Some of the known risk factors for native valve endocarditis are pre-existing valvular heart disease such as rheumatic heart disease, male sex, age > 60, congenital heart disease, structural heart disease such as cardiomyopathy, poor dental hygiene or dental infections, intravenous drug abuse, implantable cardiac device including pacemakers, and history of endocarditis or valvular heart surgery. IE most commonly affects the mitral valve, followed by the aortic valve. Culture-negative endocarditis accounts for around 5-10% of all cases of endocarditis and poses a clinical challenge to diagnose and treat; if left untreated, it carries a particularly poor prognosis [6].

Prosthetic valve endocarditis (PVE) is seen in 1-6% of all patients with prosthetic valves, with an annual incidence of 0.3-1.2% per patient-year [7]. PVE has an equal incidence in both mitral and aortic valves. PVE is further classified as early or late depending on the timing of the onset of endocarditis in relation to the surgery. Studies demonstrate that the risk of endocarditis is higher in bioprosthetic valve when compared to mechanical valves [8]. The incidence of PVE in surgical aortic valve replacement is similar to endocarditis in patients who underwent transcatheter aortic valve implantation (TAVI) [9].

Though TTE is the initial investigation conducted to evaluate the cardiac valvular function, TEE is more sensitive and specific for diagnosing vegetations, abscess, fistula, paraprosthetic valvular leak, or pseudoaneurysm. There are many well-known complications of IE, of which systemic embolization and systemic infection (vegetation/pus from the abscess) are common.

Heart failure is the most common cause of mortality from IE, followed by perivalvular abscess. Patients with perivalvular abscess are twice as likely to suffer from systemic embolization when compared to IE without an abscess. Mortality from IE is more common in patients with a perivalvular abscess [10,11]. Septic pulmonary emboli can occur in patients with right-sided valvular IE, whereas other systemic embolization occurs in patients with left-sided valvular IE and patients with right-sided valvular IE with a right-to-left shunt such as a patent foramen ovale [12].

Some of the known risk factors for embolization in IE are left-sided IE (mitral more common than aortic), large vegetation (>10 mm), advancing age, concurrent atrial fibrillation, diabetes mellitus, and hypercoagulable state such as anti-phospholipid antibody syndrome [13,14]. Systemic septic emboli from IE are more common to the brain, followed by the spleen, lungs, kidneys, peripheries, heart, and mesentery [15]. Septic emboli to the spleen result in splenic infarction or abscess or hemorrhage, and also the spleen the most common abdominal organ affected in IE [16]. Septic emboli to the mesentery occur due to occlusion of the SMA, and the resultant bowel ischemia is successfully treated by removing that portion of the bowel which is necrotic and gangrenous [17].

Mesenteric ischemia has a remarkably high in-hospital mortality rate due to its non-specific signs and symptoms at presentation which leads to delay in diagnosis. The SMA is usually the culprit vessel due to its small take-off from the aorta, and the common site of the emboli is usually distal to the origin of the middle colic artery [18,19]. Acute mesenteric ischemia should be always kept in mind while evaluating patients with IE. Cause of death in such patients is usually bowel necrosis and septic shock.

Embolic events can be prevented by appropriate initiation of antibiotic treatment, but this is not always the case as the patient might present with an embolic event as the first clinical presentation of IE. This is sometimes tricky in patients presenting with culture-negative endocarditis. If septic emboli are not identified or treated early, it can precipitate a mycotic aneurysm, which is a rare complication seen in patients with IE [20]. Multiple systemic emboli or septic emboli to multiple organs in IE are exceedingly rare and only a few cases have been reported.

## Conclusions

DKA can be a deceiving presentation of an underlying serious etiology. A bedside TTE and TEE are helpful for initial assessment of patients. Appropriate tests must be carried out on patients labelled as type 2 diabetes mellitus presenting with recurrent DKA to ensure they do not have LADA, where the patient needs to be treated with an appropriate insulin regime, rather than oral hypoglycemics. If vegetations or root abscess are visualized in the presence of vague abdominal symptoms, a computed tomography (CT) scan of the chest, abdomen, and pelvis should also be performed to rule out any embolic events that may increase the morbidity and mortality of patients with IE.
